# Kin discrimination and possible cryptic species in the social amoeba *Polysphondylium violaceum*

**DOI:** 10.1186/1471-2148-11-31

**Published:** 2011-01-27

**Authors:** Sara E Kalla, David C Queller, Andrea Lasagni, Joan E Strassmann

**Affiliations:** 1 Department of Ecology and Evolutionary Biology, Rice University, MS-170, 6100 Main Street, Houston, TX 77005, USA

## Abstract

**Background:**

The genetic diversity of many protists is unknown. The differences that result from this diversity can be important in interactions among individuals. The social amoeba *Polysphondylium violaceum, *which is a member of the Dictyostelia, has a social stage where individual amoebae aggregate together to form a multicellular fruiting body with dead stalk cells and live spores. Individuals can either cooperate with amoebae from the same clone, or sort to form clonal fruiting bodies. In this study we look at genetic diversity in *P. violaceum *and at how this diversity impacts social behavior.

**Results:**

The phylogeny of the ribosomal DNA sequence (17S to 5.8S region) shows that *P. violaceum *is made up of at least two groups. Mating compatibility is more common between clones from the same phylogenetic group, though matings between clones from different phylogenetic groups sometimes occurred. *P. violaceum *clones are more likely to form clonal fruiting bodies when they are mixed with clones from a different group than when they are mixed with a clone of the same group.

**Conclusion:**

Both the phylogenetic and mating analyses suggest the possibility of cryptic species in *P. violaceum*. The level of divergence found within *P. violaceum *is comparable to the divergence between sibling species in other dictyostelids. Both major groups A/B and C/D/E/F show kin discrimination, which elevates relatedness within fruiting bodies but not to the level of clonality. The diminished cooperation in mixes between groups suggests that the level of genetic variation between individuals influences the extent of their cooperation.

## Background

Identifying cryptic species is important; morphological similarity may mask great differences in physiology, ecology, and behavior [1]. For example, *Oreaster reticulatus *starfish preferentially prey on only one of two sympatric cryptic species of Caribbean fire sponges (*Tedania ignis *and *T. klausi*) [2]. Sympatric cryptic species of African weakly electric fishes (*Campylomormyrus tamandua *and *C. numenius*) exhibit different patterns of electric organ discharge that these fishes use for both electrolocation and communication [3]. In these cases, identifying the species has led to a greater understanding of the variation in these traits. In African weakly electric fishes, this variation in communication affects interactions between individuals such as mate recognition and mate choice.

Social behavior can be doubly impacted by cryptic speciation. In addition to differences in behavior between the two species, social interactions are dependent on the relationship between the interactors. If the interactors come from different species, then the individuals should be much less likely to perform altruistic acts. For example, in the two parapatric wood ant cryptic species *Formica lugubris *and *F. paralugubris*, workers exhibit discrimination against brood from the other sibling species when the workers are returning exposed brood to the nest [4]. However, the two species do not always discriminate against brood that is from the same species but from a different nest [4].

**Figure 1:**
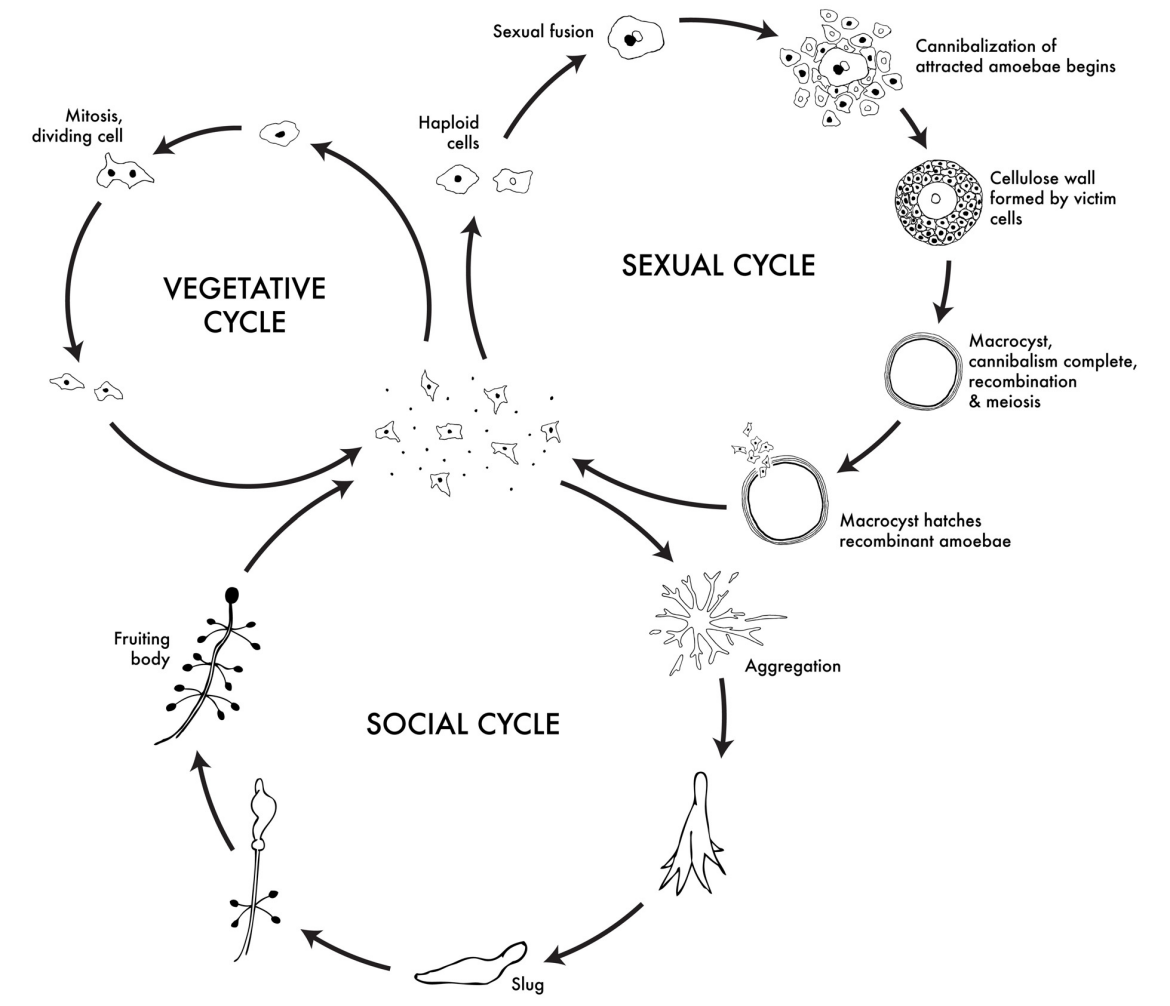
**Life cycle of *Polysphondylium violaceum***. Most of its life, this haploid social amoeba undergoes the vegetative cycle, preying upon bacteria in the soil, and periodically dividing mitotically. When food is scarce, either the sexual cycle or the social cycle begins. Under the social cycle, amoebae aggregate to glorin by the thousands, and form a motile slug, which moves towards light. Ultimately the slug forms a fruiting body in which some of the cells die to lift the remaining cells up to a better place for sporulation and dispersal. Under the sexual cycle, amoebae aggregate to glorin and sex pheromones, and two cells of opposite mating types fuse, and then begin consuming the other attracted cells. Before they are consumed, some of the prey cells form a cellulose wall around the entire group. When cannibalism is complete, the giant diploid cell is a hardy macrocyst, which eventually undergoes recombination and meiosis, and hatches hundreds of recombinants. Not drawn to scale. Image credit: David Brown and Joan Strassmann, under Creative Commons Attribution Share-Alike 3.0 license.

Dictyostelids, or social amoebae, have a complex life cycle that includes social behavior and altruism at a certain stage in their life history (Figure 1). They are unicellular haploid eukaryotes that live in soil and consume bacteria. When their food source is depleted, they aggregate together into a mound of cells, which then proceeds along one of two different forms of development. In the sexual cycle, two cells of compatible mating types fuse to form a giant cell where the nuclei fuse and undergo meiotic recombination [5,6] (Figure 1). The giant cell engulfs surrounding cells and eventually encysts. In nature haploid, recombined daughter cells eventually hatch from the cysts, though this is not easily achieved in the laboratory [7]. In the social stage, the aggregation organizes into one or more multicellular slugs. These slugs then develop into fruiting bodies. During fruiting body formation, some of the cells die to form a stalk and other cells form hardy spores at the top of the stalk. Because stalk cells die, they should be expected to preferentially form fruiting bodies with identical or closely related clones and discriminate against individuals and non-kin by sorting from them and forming independent, clonal, fruiting bodies.

There have been several studies of the diversity of individual species of dictyostelids [5,8-11], but overall there has been little study on the diversity within any one species. It has been suggested that *Polysphondylium violaceum *is a cryptic species complex composed of at least two separate morphologically identical species [5,11]. Clark examined 49 clones of *P. violaceum *collected in Massachusetts for macrocyst formation. Clark observed two different groups within *P. violaceum *that each formed macrocysts when individuals from the same group were paired [11]. She did not further characterize the two putative species, and the clones are not available for further study.

Kin discrimination has been observed in *D. discoideum, D. giganteum*, and *D. purpureum *[12-14], but no such work has been done with *P. violaceum*. It differs from the previously studied species in its branching fruiting body with many small clumps of spores [15], and it is basal to group 4 dictyostelids [16]. It is not phylogenetically close to most other species that were also called *Polysphondylium *because they were classified on the basis of their branched fruiting bodies, and not phylogenetics [13].

We examined cryptic speciation and kin recognition in *P. violaceum*. We used both DNA sequence data and mating data to look at both the population structure of *P. violaceum *and how this structure relates to cooperation in the social stage. We sequenced ribosomal DNA of 90 clones of *P. violaceum *and constructed a gene tree to examine population structure. We also performed mating experiments to understand patterns of potential gene flow. We tested for cooperation and discrimination by performing 13 mixes of cells from pairs of clones, that were then allowed to develop into the social stage, so sorting could be investigated.

## Methods

### A. Collection of clones

We collected 80 clones from undisturbed areas of the Houston Arboretum and Nature Center, Houston, TX (27 clones); Brazos Bend State Park, Needville, TX (1 clone); Mountain Lake Biological Station, Mountain Lake, VA (16 clones); Linville Falls, NC (1 clone); Urbana, IL (22 clones); and Heidelberg, Germany (9 clones) (see additional file 1 for details). *P. violaceum *is a cosmopolitan species, found throughout the world including the Americas [17-19], Europe [20,21], Asia [22-24], and Australia [25].

To ensure that each sample had only one genotype, we clonally isolated *P. violaceum *from soil samples. We cultured them on hay infused agar plates (1 L hay infused H_2_O (15 g hay left in 1.5 L H_2_O overnight, then filtered), 1.5 g KH_2_PO_4_, 0.62 g Na_2_HPO_4_, 15 g agar, autoclaved) with *Klebsiella aerogenes *as a food source. We then replated them so that individual cells grew into discrete colonies. We harvested one colony from each sample to ensure the clones were clonal. Additional file 1 has a complete list of the clones. We obtained five clones from South Africa (RSA clones) from J. Landolt and acquired the clone P6 and four clones from Wisconsin (WS clones) from the Dictybase stock center (P6 depositor: P. Schaap, WS clones depositor: G. Erdos, [26]). Initially, we used the morphology of fruiting bodies to identify clones as *Polysphondylium violaceum*. *P. violaceum *has a unique fruiting body structure, with each stalk supporting multiple whorls of spore containing sori (3-5 sori per whorl) at regularly spaced intervals and a solitary sori at the end. The sori range in color from lavender to violet (for a complete description, see [15]).

### B. Genetic analysis

To look at the relationships between wild clones of *P. violaceum*, we sequenced a ~2500 bp region that included the 17S, internal transcribed spacer 1, and 5.8S RNA (17S-5.8S) of each clone. The 17S rDNA sequence has already been used to look at the phylogeny of the entire group of dictyostelids [16,27] as well as the population structure within *D. purpureum *[9] and *D. giganteum *[7]. This sequence has enough resolution to distinguish between sister species in the dictyostelids [16,27], and prior work with this sequence gives us information on the level of divergence among species accepted as different. We used the sequences to construct a gene tree of all wild clones. The sequences of the primers we used are listed in Table 1. These primers were previously used for phylogenetics in *D. purpureum *and *D. giganteum *[8,9].

**Table 1:** Primers used in sequencing the 17S and 5.8S region of ribosomal DNA in *P. violaceum*

Name	Sequence 5'-3'
Sandie_A	AACCTGGTTGATCCTGCCAGT

17S_r1	AGATAATACAAGCTGAACTA

17S_f2	GCTCGTAGTTGAAGTTTAAG

1340_r	TCGAGGTCTCGTCCGTTATC

17S_f3	CTAAGATATAGTAAGGATTG

17S_r3	ATGATCCATCCGCAGGTTCA

ITS_5.8_f1	ACGGTAAAGTTAACG GATCG

ITS_5.8_r1	ACTCTCACCCAAGTATAACT

ITS_5.8_f2	AAACTGCGATAATTCACTTG

ITS_5.8_r2	CCGTCTTCACTCGCCGTTAC

We harvested DNA by collecting 5-10 fruiting bodies into 150 μl of a 5% Chelex solution (Bio-Rad, Hercules, CA, USA), then added proteinase K to a concentration of 1.25 mg/ml and incubated this solution at 56°C for four hours then 98°C for 30 minutes.

We amplified this region with a polymerase chain reaction using Invitrogen's Platinum taq polymerase and 0.5 μM of each primer, using chelexed DNA as template. PCR cycling conditions were as follows: initial denaturation at 94°C for 5 min, followed by 30 cycles of 1 min denaturation at 94°C, 1 min annealing at 50°C, 1 min elongation at 72°C, followed by a final elongation at 72°C for 10 min. We sequenced all PCR products in both directions using Big Dye Terminators (Applied Biosciences, Foster City, CA, USA) and analyzed with an ABI Prism automated sequencer (Applied Biosciences, Foster City, CA, USA). We edited chromatograms and aligned contigs using the programs SeqMan (DNASTAR, Madison, WI, USA) and BioEdit (Hall, http://www.mbio.ncsu.edu/BioEdit/bioedit.html). Sequences have been deposited in Genbank [HQ732139-HQ732228].

We followed procedures previously used in our group for phylogenetic analyses [9]. We included as outgroups *D. purpureum *and *D. citrinum *which are two group 4 dictyostelids (Genbank: *D. purpureum *DQ340386.1, *D. citrinum *DQ340385.1). We aligned sequences using ClustalW [28]. We developed a gene tree using Bayesian inference (Mr. Bayes, [29]). To determine the optimal nucleotide substitution model, we used Akaike information criteria (AIC) [30] and Bayesian information criteria (BIC) [31], as implemented in ModelGenerator [32]. A Generalized Time Reversible Model with a gamma distribution of mutations (GTR + Γ) was found to be the best model according to both AIC and BIC (data not shown). We used Mr. Bayes [29] to construct a gene tree and to estimate posterior probabilities for each node with parameters estimated based on the model recommended by ModelGenerator [32], the GTR + Γ model. The program ran four Metropolis-Coupled Markov chains for 1,600,000 generations following a burn-in period of 400,000 generations with sampling every 100 generations and beginning with a random tree. We looked at the average standard deviation of split frequencies to check convergence. By the 10,000 sampled tree, the average standard deviation of split frequencies had stabilized at ~0.011, and did not decrease in the following 6,000 sampled trees. We also checked convergence with Are We There Yet? (AWTY, http://ceb.scs.fsu.edu/awty) [33]. We used the 'compare' option to compare the posterior probabilities of clades from independent runs checking to make sure that the posterior probabilities of the splits are the same for both independent runs. Nodes with posterior probabilities of less than 0.80 were collapsed.

We also generated a Maximum Likelihood tree using Garli [34]. We generated 500 bootstrap replicates. We used consense to generate a consensus tree with bootstrap support. Seqboot, dnaml, and consense are all part of the Phylip package [35].

### C. Macrocyst formation experiments

When dictyostelid cells aggregate in response to starvation, there are two developmental pathways that the cells can take - the social, fruiting body stage or the sexual macrocyst stage as shown in Figure 1. Macrocyst formation is favored when cells are cultured in the dark, under liquid, and without phosphate [6]. During macrocyst development, amoebae are attracted to the cAMP produced by the diploid fusion of two cells of different mating types. The attracted amoebae wall themselves in and are gradually consumed by the sexual cell which forms a giant cell that divides many times before germination when they release hundreds of recombined amoebae [6]. Clones of the opposite sex and same species form macrocysts under appropriate conditions, though it is extremely difficult to get these macrocysts to hatch in the laboratory. This means that macrocyst formation is only a partial test for true sexual compatibility.

To test for macrocyst formation, we incubated each clone both by itself and with each other clone tested under conditions favorable for macrocyst formation. We tested clones for macrocyst formation by plating spores on phosphate-free lactose peptone agar (1 g lactose, 1 g bactopeptone, 15 g agar, 1 l diH_2_O) with *K. aerogenes *as a food source. We then flooded these plates with Bonner's standard salt solution (5.4 mM CaCl_2_, 10 mM KCl, 5.1 mM NaCl), wrapped them in aluminum foil, and incubated in the dark for 3-5 days. After 5-7 days, we scored macrocysts as either present or absent for each treatment. When checked at later times (2-3 checks within 3-5 weeks) no additional macrocysts had formed. In mixes where no macrocysts had formed, cells usually reached aggregation stage and stopped or the cells simply died. In a few cases, cells made fruiting bodies or spores. For most mixes, the clones were divided into sets and all combinations of clones were mixed within that set. The sets were then replicated twice. If both clones were in two different sets, then that particular mix was performed 4 times (for example, QSvi9 and QSvi29).

### D. Testing kin discrimination

To test for kin discrimination, we performed 13 reciprocal pairwise mixes. For each mix, we fluorescently labeled two clones, and mixed each clone with unlabeled cells of the same clone and unlabeled cells of the other clone. We performed both the reciprocal mixes, to control for any effects of labeling, and mixes within the same clone to ensure that the cells were healthy. We allowed these four mixes to starve, aggregate and form fruiting bodies. We followed the same protocol as [13].

Cells of each clone were grown up to log phase, split into two groups, one of which was labeled with 5-chloromethylfluorescein diacetate (CellTracker TM, Invitrogen, Carlsbad, CA, USA). These cells were then mixed together in the following fashion: labeled cells of the first clone mixed with unlabeled cells of the first clone, labeled cells of the second clone mixed with unlabeled cells of the second clone, labeled cells of the first clone mixed with unlabeled cells of the second clone, and labeled cells of the second clone mixed with unlabeled cells of the first. Additionally, we plated out the labeled and unlabeled cells of each clone alone as controls. We collected individual fruiting bodies from each treatment and counted the number of fluorescently labeled spores and the number of unlabeled spores to determine the proportion of each clone present in the fruiting body.

### E. Statistics

To evaluate the extent of sorting in each fruiting body we calculated the average relatedness of the spores in each fruiting body assuming that each clone was completely related to itself (r = 1) and completely unrelated to the other clone (r = 0). Relatedness of the overall fruiting body is calculated as the proportion of labeled cells or spores squared plus the proportion of unlabeled cells or spores squared (r = p^2^+q^2^); that is, p of the cells are related by p to the other cells, and q of the cells are related by q. We calculated relatedness individually for each fruiting body and then averaged over all fruiting bodies. We measured sorting as a significantly higher relatedness in the experimental fruiting bodies than in control fruiting bodies.

Because the data were not normally distributed, we used Resampling Stats for Excel (Resampling Stats Inc., Arlington, VA, USA) to create a test. We calculated the test statistic [F = Variance (experimental)/Variance (control)] as the ratio of the average variance of the two experimental treatments divided by the average variance of the two control treatments. We sampled without replacement the dataset of the proportion of fluorescent spores of each individual fruiting body across the four treatments (two experimental and two control) 5000 times to determine the probability that a variance ratio as high as this observed ratio could be obtained by chance [13].

To test for geographic population structure, we ran an Analysis of Molecular Variance (AMOVA) using Arlequin 3.11 [36] and used resampling (1023 permutations) to obtain significance values.

## Results

### Sequence analysis and phylogeny

**Figure 2:**
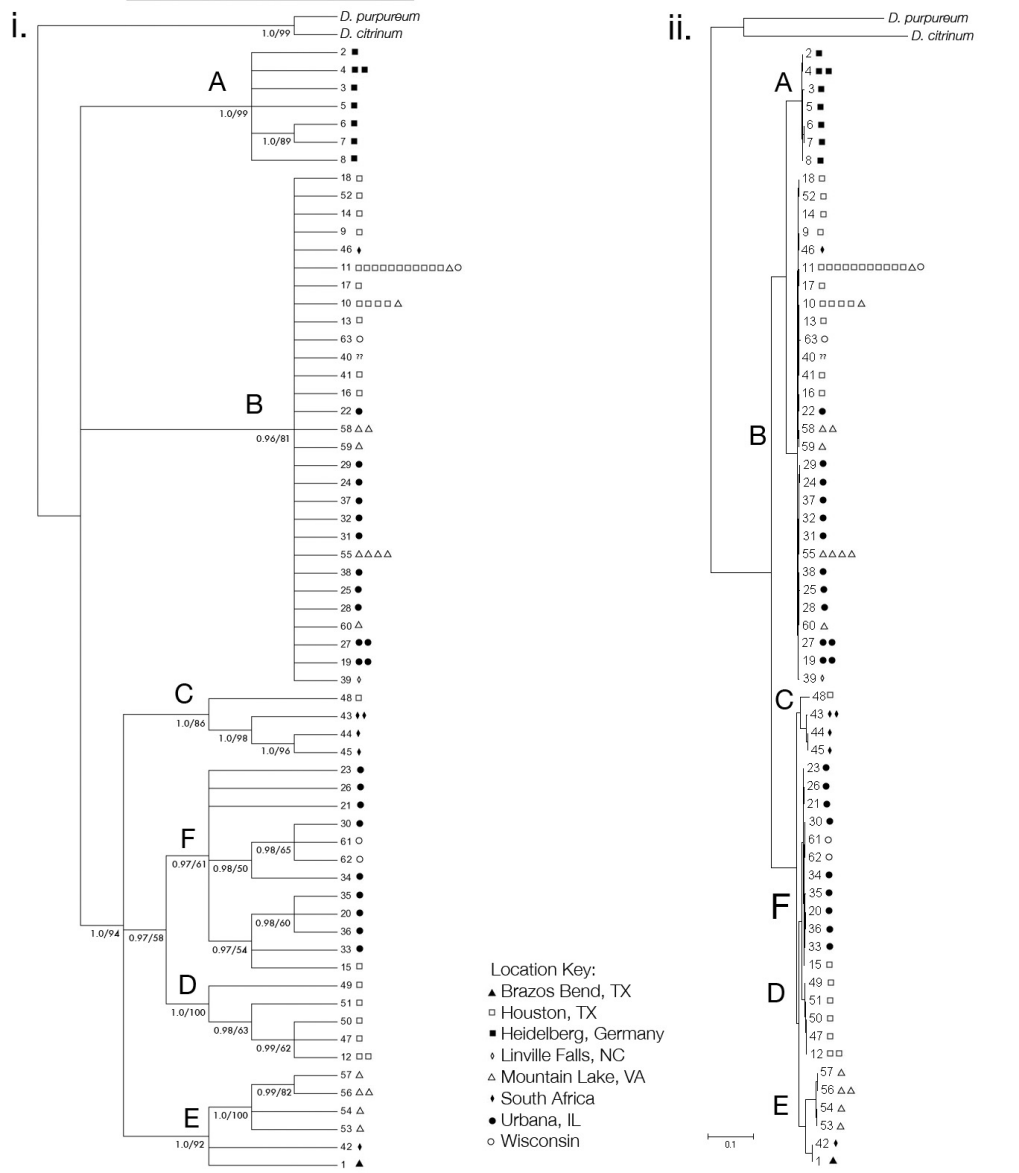
**Bayesian gene tree based on ~2400 bp from 17S-5.8S RNA region of the ribosome of *P. violaceum *clones**. *Dictyostelium purpureum *and *D. citrinum *were used as the outgroups. Each symbol represents one clone, and each branch represents one unique haplotype. The letters simply refer to different phylogenetic groups. i. Cladogram with nodes with Bayesian inference posterior probabilities of less than 0.95 collapsed. Numbers on the nodes are the Bayesian posterior probabilities and bootstrap values from the maximum likelihood analysis. ii. Phylogram.

We sequenced approximately 2500 bp of the 17S to 5.8S ribosomal DNA for 90 clones of *P. violaceum*. Out of the 90 clones sequenced, we identified 67 unique haplotypes. We aligned these sequences and used the two species *D. citrinum *and *D. purpureum *as outgroups. The resultant Bayesian gene tree is shown in Figure 2 with the support values from both the Bayesian gene tree and the maximum likelihood gene tree shown on the tree in Figure 2i.

We find that the *P. violaceum *is split into six major groups, labeled A, B, C, D, E, and F on the phylogeny (Figure 2). Groups C, D, E, and F made up one of the basal branches of the phylogeny while the group A and group B made up the other two branches. The phylogeny shows some evidence for geographic population structure. Group A is comprised of all of the clones from Germany. All of the clones in group D were from Houston, TX, however Houston clones belonged to other groups as well. Most of the clones in groups C and E were from the same location (South Africa and Mountain Lake, VA respectively), however these locations also had clones that belonged to other groups. Not all phylogenetic divisions came from geographic structure. Clones in group B came from 6 of the 8 locations that we sampled.

Using a shorter sequence to enable the use of *D. laterosorum *as an outgroup results in a tree that is similar to the tree resulting from the full length sequence (Additional file 2). Groups A and B still form a branch together. The node with groups C, D, E and F has been collapsed to a polytomy. All but one of the haplotypes in group C cluster as a group still. All but 2 haplotypes in group E cluster as a groups still. Group D remains unchanged. Group F has been collapsed entirely, and the groups C, D and E no longer show any relationship with one another.

To see if population structure was due to geographic distance, we calculated the Analysis of Molecular Variance (AMOVA) using the 17S to 5.8S sequence. Like Fst, the AMOVA is a measure of the population variance, however the AMOVA also incorporates the degree of difference (mutations) between alleles [37]. The AMOVA showed that 7.75% of the genetic variation observed was between geographically delimited populations, and the variance was significant (p < 0.00001).

To look at the level of divergence between the phylogenetic groups, we used the 17S sequence to calculate pairwise distances (base substitutions per site) between all of the clones. We used MEGA4 to calculate the distances using the Maximum Composite Likelihood method [38,39]. Using just the 17S sequence, we calculated the average pairwise distance between clones of *P. violaceum *and *D. laterosorum *(clone AE4). These distances ranged from 0.013 to 0.019 (data not shown). Between the two major groups (A/B and C/D/E/F), pairwise distances ranged from 0.010 to 0.021 (data not shown). Within these two major groups, distances ranged from 0.000 to 0.010. Within each of the six groups, the maximum pairwise distances ranged from 0.000 (groups B, C, and F) to 0.010 (group E). Within groups, all clones had a minimum pairwise distance of 0.000. The average pairwise distances between some clones are analogous to the average pairwise distances between clones of *P. violaceum *and *D. laterosorum*, which are clearly different species. This suggests that *P. violaceum *has species level diversity.

### Macrocyst matings

To further look at speciation in *P. violaceum*, we also examined macrocyst formation (Table 2). No individuals from group A had any successful matings, either with other members of group A or members of other groups. In group B, we observed two different mating types: we had two clones of one mating type and 23 clones of the other mating type. With one exception, all matings between clones of compatible mating types resulted in macrocyst formation. In the other groups C, D, E, and F, the mating types were not as clear because of triads of clones where all three clones would mate with each other in pairwise combinations, leading to uncertainty about the number of sexes and whether clones might be bisexual. Groups D and E both had two clearly defined mating types, and each clone made the sexual form called a macrocyst when paired with a member of the same group but opposite mating type. One clone from group E also mated with group C, though group C did not mate at all within itself, perhaps because our sample did not include compatible sexes. Clones from group F mated between themselves as well as with clones from other groups. We performed multiple replicates of each set of mixes. While most mixes were consistent between replicates, some mixes (16 out of 835 mixes) formed macrocysts only in some of the replicates. In these cases, the result (macrocyst formation or no macrocyst formation) that was found in the majority of replicates was used, to rule out the possibility of contamination or occasional selfing.

**Table 2:** Macrocyst production in *P. violaceum*

	A	B	C	D	E	F
A	0/21	0/24	0/1	0/7	0/7	0/3
B		46/287	1/101	0/75	1/33	5/87
C			0/6	0/26	4/17	2/14
D				6/12	1/22	2/13
E					3/6	0/7
F						16/66

The letters refer to the phylogenetic groups from Figure 2. A total of 835 unique mixes were attempted between two clones. In each category, matings are listed as successful mixes/attempted mixes. Each mix between any two individuals was performed at least twice. When these gave conflicting results, another replicate mix was performed and the majority result was counted. The individual clones used, as well as their haplotype and their group on the phylogenetic tree are listed in the Additional file S1.

Overall, we saw complete mating within group B (all clones of mating type A mated with all of mating type B), and a high degree of mating within the other major clade of groups C, D, E, and F, with a few matings between two major clades. Between the groups C, D, E, and F there was some mating between the different groups though matings were not consistent enough to be able to assign mating types to clones and thus diagnose the thoroughness of mating success.

We are hesitant to categorize the rate of successful matings between because of the variation in mating types throughout the dictyostelids. *D. discoideum *has at least two different mating types, with a bisexual mating type in addition [40]. In *D. rosarium*, at least three mating types are present [41]. Group B of *P. violaceum *had two well-defined mating types, and all clones mated when paired with a member of the opposite mating type of Group B. However, we were unable to identify the number of mating types within the other groups of *P. violaceum*. Because of this, we are not confident in estimating the number of matings that could occur based on mating types and this is necessary to compare the rates of observed matings to the possible matings.

### Kin discrimination

**Figure 3:**
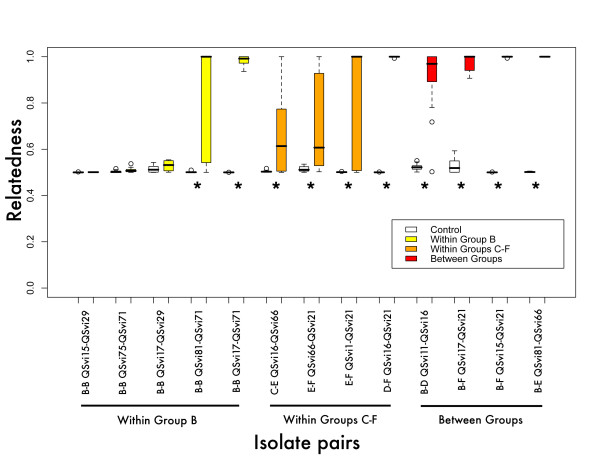
**Box and whisker plot of relatedness of individual clones in fruiting bodies**. Relatedness is calculated as the proportion of labeled cells squared plus the proportion of unlabeled cells squared (r = p^2^+q^2^), which assumes r = 1 to clonemates and r = 0 to non-clonemates. Relatedness was calculated for each fruiting body individually. Relatedness varies from 0.5 (complete mixing) to 1 (sorting). Groups refer to the six lettered groups on the phylogenetic tree. Resampling stats were used to compare the relatedness of the control mix (labeled and unlabeled cells of the same clone) to the experimental (labeled and unlabeled cells from different clones). Error bars are standard error of the mean. * the control mix relatedness is lower than the experimental p < 0.001. See methods for details.

We looked at the influence of phylogenetic relationships between individuals on cooperation. To do this, we related genetic distance to the degree of sorting between clones. Both distantly related clones and clones from different species should be less likely to cooperate in forming fruiting bodies. All but three pairwise mixes showed significant sorting in comparison to controls (Figure 3, Resampling stats). These three mixes were all between members of group B (Figure 3). While the rest of the mixes were all statistically significant from the controls, some of the mixes between groups C-F showed incomplete sorting (relatedness < 1). The average relatedness of the pairwise mixes was 0.8 (0.05 std error).

## Discussion

Our data suggest that *P. violaceum *might contain cryptic species. There are 6 groups (A, B, C, D, E, and F) with the most basal split being between the groups A/B and C/D/E/F. Though the genetic distances between these two main groups are relatively small (0.010-0.021), they are larger than the differences between accepted species in the dictyostelids using the same part of the 17S sequence, for example between *D. citrinum *(OH494) and *D. dimigraformum *(AR5b), 0.009; *D. clavatum *(TNS-C-189) and *D. longosporum *(TNS-C-109), 0.003; *D. mucoroides *(TNS-C-114) and *D. sphaerocephalum *(GR11), 0.001 or between *D. brunneum *(WS700) and *D. giganteum *(WS589) 0.004 [9]). The 17S to 5.8S sequence is relatively conserved compared to other genes in a wide variety of organisms (for example [42-44]). This sequence has enough resolution to distinguish between sister taxa in the dictyostelids [16,27].

Furthermore, the variation between geographical populations accounts for less than 10% of the total variation. Many haplotypes are found in more than one geographic population. In addition, group B is cosmopolitan, with individuals from almost every geographic location belonging to this group. This suggests that the population structure in *P. violaceum *is not due to geographic constraints alone. The major differences that we see in the 17S seem for the most part consistent with species-level differences, with the species often occurring in the same areas.

By and large, the division between the two groups was reinforced by our mating experiments. Like Clark [5,11], we found two groups of non-interbreeding individuals; however, we observed a few instances of mating between the groups. This leaves open the possibility for some gene exchange between different groups should those macrocysts actually be able to germinate. Unlike the model organism, *D. discoideum*, *P. violaceum *reported germination rates have been upwards of 50% [45]. Examining the germination of our between-group macrocysts, which would require prolonged ageing of macrocysts [45], would be a fruitful line of research for the future.

The split between the two groups is also apparent when looking at cooperation during fruiting body formation. We have found that only clones from group B exhibit strong mixing and cooperation with other group B clones in forming the fruiting body. When the clones were from different phylogenetic groups sorting was more complete. Both the phylogenetic diversity and the behavioral changes suggest that there may be at least two different morphologically identical sister species in *P. violaceum*. Both lines of evidence are consistent with the same division and the variation that we observed in phylogenetic structure affects the behavior that we observe in the social stage.

Relatedness allows altruism to be beneficial if the altruistic acts are directed towards relatives. Because clones from two different species are not related, there should be selection for species discrimination. Because we are unsure of the exact nature of the relationship between individual clones, we use the term kin discrimination rather than species discrimination. In *D. discoideum*, the further the genetic distance between clones, the greater the propensity for kin discrimination to occur [12]. In our study, a few clones cooperated with each other to form chimeric fruiting bodies, but most clones tested sorted out to form mostly clonal fruiting bodies. All the clones that cooperate with each other were in the same group (B). This fits with the idea of kin selection, with only closely related clones cooperating, though we do not have information on exact values for the other half of kin selection: the relative costs and benefits of cooperation. Benefits of larger groups are likely to include lower proportions of cells destined for stalk relative to spore, and ability to move greater distances, while costs center on becoming a sterile stalk cell.

Previous studies on kin discrimination in the dictyostelids have given differing results depending on the species used. Kin discrimination has also been investigated in *Dictyostelium discoideum*, *D. purpureum *and *D. giganteum*. Clones of *D. discoideum *exhibit kin discrimination with more distantly related clones sorting more than clones that are more closely related [12]. *D. purpureum *shows kin discrimination as well [13]. In *D. giganteum*, some genetically distinct clones exhibit kin discrimination while others do not [14]. The question of whether *D. giganteum *is one species worldwide with varying levels of kin discrimination or multiple cryptic species has not been resolved, but North American clones show no differentiation [8]. Our results show that *P. violaceum *exhibits kin discrimination; like the other dictyostelids, clones from different cryptic groups within *P. violaceum *sort to form clonal fruiting bodies while closely related clones sometimes cooperate to form chimeric fruiting bodies.

Most of the dictyostelids have been identified and distinguished on the basis of morphology. An exception is recent work on *Polysphondylium pallidum *and its sister species *P. album *[46] as well as *D. ibericum *[47]. Romeralo, Baldauf, and Cavender [47] used morphology to identify a new species, and molecular phylogenetics to place that species within the dictyostelids. Kawakami and Hagiwara [46] use a combination of mating type and morphological characters to redefine these two species. They show that there are three groups, one that matches the *P. pallidum *type specimen and mates with *P. pallidum *strains, one that matches the *P. album *type specimen and mates with *P. album *strains, and one that matches neither exactly and mates with neither. The relationship of the third group to *P. pallidum *and *P. album *remains unclear. These recent studies make it clear that relying on morphology alone to dictate species boundaries is not sufficient, and mating type analysis and molecular work is needed to correctly identify species boundaries and the relationships between species.

Improperly identifying cryptic species also affects biodiversity metrics as well as estimates of geographical distributions. By identifying all members of a cryptic species complex as the same species, biodiversity is underestimated and geographic distributions are overestimated. In the identification of protists this can be especially difficult because of a lack of distinguishing morphological characteristics [48]. The difficulty of correctly identifying cryptic species has contributed to debate on protist biogeography. Finlay [49] suggests that there is something fundamentally different about microorganisms, including protists, such as higher rates of migration and lower rates of speciation that causes them to be more cosmopolitan than larger organisms. Foissner [50] suggests that more endemic species are present in part because of molecularly distinct but morphologically similar species that are endemic but are classified as a single cosmopolitan species.

## Conclusion

Molecular sequence data has identified many cryptic protist species. Most of the cases involve apparently cosmopolitan species that are actually comprised of geographically restricted cryptic species [50]. However, this is not always the case. *Aspergillus fumigatus *is composed of several cryptic species, but rather than being geographically isolated species, at least one species is globally distributed [51]. Similarly, phylogenetic analyses divide the desert truffle *Terfezia boudieri *into 3 morphologically identical groups [52]. All collections were made in a roughly 50 km^2 ^region of the Negev desert. While these 3 species are endemic, they do have overlapping ranges, and there is the possibility that all 3 might have the same range. In addition, the ectomycorrhizal fungus, *Tricholoma scalpturatum *shows two distinct groups [53]. Roughly half of the genetic variance was found within populations and half of it was found to be between populations. At short ranges, populations were sometimes structured, but both groups were represented over the entire sampling range (France to Sweden). These previous studies suggest that cryptic species are not always endemic subgroups of a cosmopolitan morphotype. If *P. violaceum *is in fact composed of two morphologically indistinguishable species that are globally distributed, they support Foissner's [50] idea that while globally distributed cosmopolitan species exist, morphologically identical endemic species are also present.

## Authors' contributions

SEK generated the sequence, performed the mating type and some of the kin discrimination experiments, analyzed the data and drafted the manuscript. JES and DCQ conceived of the study, and participated in its design and coordination and helped to draft the manuscript. AL performed some of the kin discrimination experiments. All authors read and approved the final manuscript.

## Supplementary Material

Additional file 1 **Supplemental Table S1. A list of clones used in this study**. This excel spreadsheet contains a list of all clones used in this study, their haplotype based on ribosomal DNA sequence, and the locations that they were collected from. Click here for file

Additional file 2 **Supplemental Figure S2. Bayesian gene tree based on ~1700 bp from 17S RNA region of the ribosome of *P. violaceum *clones**. Bayesian gene tree based on ~1700 bp from 17S RNA region of the ribosome of *P. violaceum *clones. *Dictyostelium purpureum*, *D. citrinum*, and *D. laterosorum *were used as the outgroups (Genbank: *D. purpureum *DQ340386.1, *D. citrinum *DQ340385.1, *D. laterosorum *AM168046.1). The tree was constructed as detailed in the methods, with the constraint that all of the outgroups had to group together. Each symbol represents one clone, and each branch represents one unique haplotype. The letters simply refer to different phylogenetic groups. i. Cladogram with nodes with Bayesian inference posterior probabilities of less than 0.95 collapsed. Numbers on the nodes are the Bayesian posterior probabilities. ii. Phylogram. Click here for file
