# Supplementary figures and images for: Kin discrimination and possible cryptic species in the social amoeba Polysphondylium violaceum
Source: BMC Evol Biol. 2011 Jan 27;11:31. doi: 10.1186/1471-2148-11-31 (PMC3041686; doi:10.1186/1471-2148-11-31)

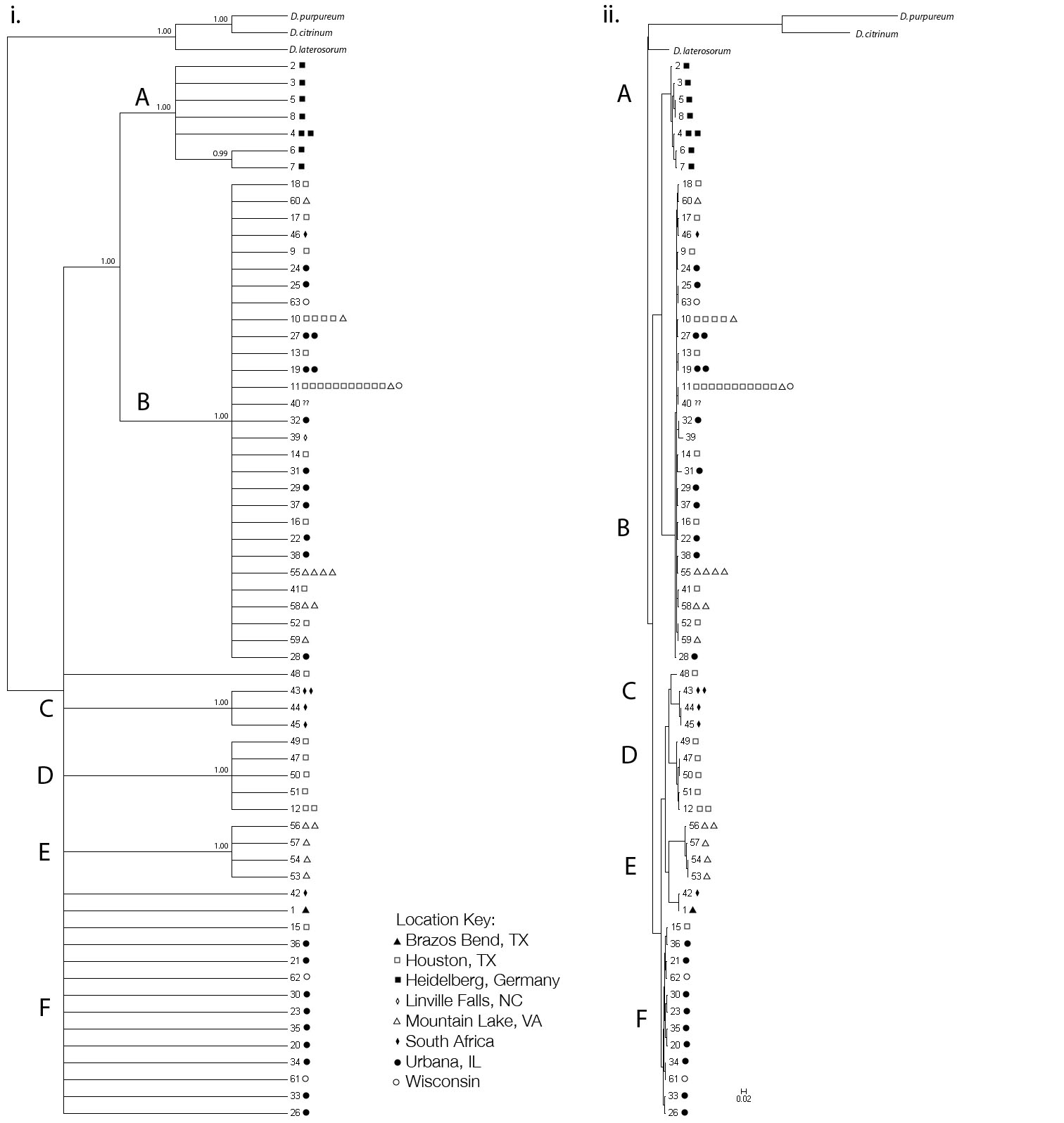

Supplement: Additional file 2 — Supplemental Figure S2. Bayesian gene tree based on ~1700 bp from 17S RNA region of the ribosome of P. violaceum clones. Bayesian gene tree based on ~1700 bp from 17S RNA region of the ribosome of P. violaceum clones. Dictyostelium purpureum, D. citrinum, and D. laterosorum were used as the outgroups (Genbank: D. purpureum DQ340386.1, D. citrinum DQ340385.1, D. laterosorum AM168046.1). The tree was constructed as detailed in the methods, with the constraint that all of the outgroups had to group together. Each symbol represents one clone, and each branch represents one unique haplotype. The letters simply refer to different phylogenetic groups. i. Cladogram with nodes with Bayesian inference posterior probabilities of less than 0.95 collapsed. Numbers on the nodes are the Bayesian posterior probabilities. ii. Phylogram. [file 1471-2148-11-31-S2.JPEG]
